# Personal Protective Equipment and Antiviral Drug Use during Hospitalization for Suspected Avian or Pandemic Influenza^1^

**DOI:** 10.3201/eid1310.070033

**Published:** 2007-10

**Authors:** Ashwin Swaminathan, Rhea Martin, Sandi Gamon, Craig Aboltins, Eugene Athan, George Braitberg, Michael G. Catton, Louise Cooley, Dominic E. Dwyer, Deidre Edmonds, Damon P. Eisen, Kelly Hosking, Andrew J. Hughes, Paul D. Johnson, Andrew V Maclean, Mary O’Reilly, S. Erica Peters, Rhonda L. Stuart, Rodney Moran, M. Lindsay Grayson

**Affiliations:** * Austin Health, Melbourne, Victoria, Australia; † St. Vincent’s Health, Melbourne, Victoria, Australia; ‡ Barwon Health, Geelong, Victoria, Australia; § Victorian Infectious Diseases Reference Laboratory, Melbourne, Victoria, Australia; ¶ Royal Hobart Hospital, Hobart, Tasmania, Australia; # Westmead Hospital, Sydney, New South Wales, Australia; ** Royal Melbourne Hospital, Melbourne, Victoria, Australia; †† University of Melbourne, Melbourne, Victoria, Australia; ‡‡ Box Hill Hospital–Eastern Health, Melbourne, Victoria, Australia; §§ Western Hospital, Melbourne, Victoria, Australia; ¶¶ Monash Medical Centre–Southern Health, Melbourne, Victoria, Australia; ## Department of Human Services, Melbourne, Victoria, Australia; *** Monash University, Melbourne, Victoria, Australia

**Keywords:** Influenza, avian, pandemic, antivirals, health resources, infection control, healthcare workers, disease transmission, research

## Abstract

For pandemic influenza planning, realistic estimates of personal protective equipment (PPE) and antiviral medication required for hospital healthcare workers (HCWs) are vital. In this simulation study, a patient with suspected avian or pandemic influenza (API) sought treatment at 9 Australian hospital emergency departments where patient–staff interactions during the first 6 hours of hospitalization were observed. Based on World Health Organization definitions and guidelines, the mean number of “close contacts” of the API patient was 12.3 (range 6–17; 85% HCWs); mean “exposures” were 19.3 (range 15–26). Overall, 20–25 PPE sets were required per patient, with variable HCW compliance for wearing these items (93% N95 masks, 77% gowns, 83% gloves, and 73% eye protection). Up to 41% of HCW close contacts would have qualified for postexposure antiviral prophylaxis. These data indicate that many current national stockpiles of PPE and antiviral medication are likely inadequate for a pandemic.

## Abstract 2

In a pandemic, many current national stockpiles of PPE and antiviral medications are likely inadequate.

Although a new influenza pandemic may appear inevitable, critical parameters of transmissibility and attack rate are uncertain. Estimates based on extrapolations from the 3 influenza pandemics of the 20th century suggest that healthcare facilities in the United States alone may be required to cope with 314,000–734,000 additional hospitalizations and 18–42 million outpatient visits (*1*). During the early containment phase of a pandemic, patients with suspected infection are likely to be referred to hospitals for isolation, diagnosis, and treatment until the transmissibility and virulence of the pandemic strain are known. Although social distancing and school closures may reduce risk in the wider community (*2*), healthcare workers (HCWs) are likely to encounter repeated close exposures. If hospitals are to continue to function adequately, reliable access to effective personal protective equipment (PPE; gowns, N95 masks, gloves, and eye protection) and antiviral drug therapy will be necessary for an unpredictable period. With awareness of the recent severe acute respiratory syndrome (SARS) outbreak and with growing concern about human deaths from avian influenza (H5N1), governments worldwide have begun to stockpile PPE and antiviral medication.

Key strategies to control the speed and extent of viral spread within healthcare settings have been advocated by national government guidelines (*3*–*6*) and the World Health Organization (WHO) (*7*). These include rigorous infection control practices, prescriptive instructions for the use of PPE, and dissemination of antiviral medication. However, information regarding the required quantity and rate of use of these valuable resources in an outbreak situation is lacking, thereby limiting valid assessments of the adequacy of current stockpiles. This study aimed to estimate the resource needs that a hospital might face in the first few hours of management of a single patient who sought treatment with possible avian or pandemic influenza (API) or similar highly virulent respiratory infection.

## Methods

In a prospective, multicenter, simulation exercise, we assessed the initial 6 hours of management of a patient (actor) who appeared for treatment at a hospital emergency department with a history consistent with API. Tertiary-level university teaching hospitals across eastern Australia were invited to participate. The inclusion criteria were willingness to join the simulation and possession of a formal local infection control protocol for the management of API that followed Australian (*3*) or WHO guidelines (*7*). The study was approved as a quality assurance project by the ethics committee at each participating site.

### Conduction of Simulation

For each of the participating hospitals, the 6-hour simulation was conducted midweek, beginning between 8:30 and 9:30 am, to avoid the busiest emergency department periods and to minimize the possibility that the care of actual patients might be compromised. The simulated patient was an actor unknown to the hospital staff, who appeared at the triage area of the emergency department and followed a prerehearsed script designed to trigger the hospital protocol for API. The standardized history included a 72-hour period of high fever, cough, shortness of breath, and severe malaise after a recent return from a Southeast Asian country. The patient reported handling unwell live poultry in a rural setting where human cases of avian influenza were known to have occurred. This standardized clinical scenario was chosen because guidelines for managing human cases of avian influenza (H5N1) form the current template for pandemic influenza case management (*4*,*5*,*7*). To heighten staff awareness of the appropriate management of an API case, each hospital organized education sessions on PPE use, infection control practices, and protocol familiarization in the 1–2 weeks before the simulation. Staff members were informed that the simulation would occur at some time during the allocated week (but not the exact day) and were instructed that hospital protocol should be followed as if it were an actual API case.

Each site had at least 3 trained infection control observers available who were familiar with using a modified version of a validated hand hygiene assessment data input tool (*8*) to accurately record potential API exposures in a standard manner. The observers were provided by the coordinating center or by the participating hospital. A principal investigator (A.S.) was present at each simulation to ensure standardization. The following 3 procedures were observed and assessed (Figure): 1) patient management through triage, emergency, radiology, and inpatient ward (including transfer between areas); 2) respiratory specimen collection, transport, and processing; and 3) cleaning of clinical areas after the suspected API patient had left the area or the simulation had been completed.

**Figure:**
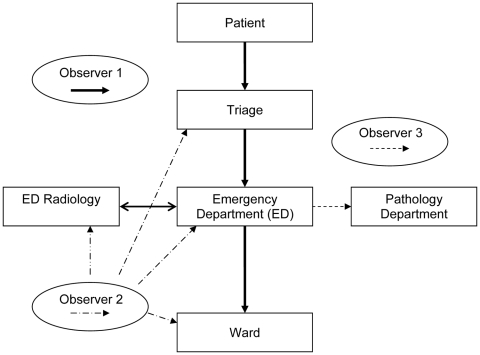
Study algorithm. Observer 1 follows the patient through all clinical areas, including transit between areas. Observer 2 monitors transport of clinical specimen to the pathology department and subsequent specimen processing. Observer 3 monitors cleaning of clinical areas after use.

Detailed observations were collated on infection control practice, clinical resources used, sequence of donning and removing PPE, time spent by the patient in each clinical area, and close contacts and exposures generated. The observation period could be stopped at any time if an actual patient’s care was judged to be compromised by continuation of the simulation.

At the time of collecting blood, respiratory specimens, or chest radiographs, surrogate specimens (venipuncture tube containing water, water-moistened swabs, and archival chest x-rays, respectively) were substituted by the accompanying study observer. Surrogate blood and respiratory specimens were followed to the laboratory, where infection control practices were observed until specimens were sent to the reference laboratory for molecular testing.

### Study Definitions

A HCW was defined as any person working within the healthcare facility. We used the WHO definition of a “close contact” as any person (including non-HCWs) coming within 1 m of an API patient within or outside of an isolation room or area (*7*). Close contacts were counted only once. An “exposure” was counted each time a close contact came within 1 m of the API patient. A “PPE item” included a disposable gown, pair of gloves, pair of protective eyewear, or N95 mask (or equivalent particulate respirator). A “PPE set” was defined as the appropriate combination of PPE items recommended for HCW use in a particular clinical setting (*7*) (Table 1). “Opportunity for PPE item use” was defined as any instance of actual use of a PPE item during the study as well as any instance where the wearing of a PPE item was recommended by WHO guidelines (*7*), as objectively noted by accompanying study observers (Table 1). These items included PPE worn by HCWs involved in direct patient care (HCW close contacts) and ancillary HCWs who performed indirect clinical tasks associated with the API case-patient such as cleaning, ward support, and specimen transportation and processing. Environmental decontamination of clinical areas after use was considered adequate if cleaning and disinfection procedures were undertaken in a manner consistent with WHO recommendations (*7*). The time spent in each clinical area was recorded from when the API patient first entered an area to the time when the patient entered the next area.

**Table 1:** WHO Recommendations for HCW barrier precautions, dependent on type of exposure*†

HCW activity	Recommended PPE set
Close contact (<1 m) with potential API-infected patient within or outside of the isolation room or area	Gloves, gown, N95 mask (or equivalent particulate respirator), eye protection
Cleaning	Gloves, either gown or apron
Patient transport within healthcare facilities	Gown, gloves
Specimen transport and processing	Not defined except to use “safe handling practices”; interpreted as use of gloves (minimum) and gown if opening specimen bag.

*WHO, World Health Organization; HCW, healthcare worker; PPE, personal protective equipment; API, avian or pandemic influenza. †Derived from (*7*).

For the purpose of identifying HCW close contacts who would be offered postexposure antiviral prophylaxis, HCW close contacts were stratified into either moderate- or low-risk groups derived from WHO criteria (*9*). High-risk close contacts, defined as “household or close family contacts of a strongly suspected or confirmed avian influenza (H5N1) patient” were not relevant to our study. The moderate-risk group included HCW close contacts wearing an insufficient or inappropriate PPE set during any of their exposures. The low-risk group included HCW close contacts wearing an appropriate PPE set for all exposures (*9*).

### Outcome Measures

The study outcome measures were the following: 1) number of close contacts associated with the API patient during the initial 6 hours of patient management, including how many of these were HCW close contacts; 2) the total number of exposures experienced by close contacts; 3) overall quantity and type of PPE items (gowns, gloves, N95 masks, eyewear) actually used during the simulation by HCW close contacts and ancillary HCWs; 4) overall “opportunities for PPE item use” for HCW close contacts and ancillary HCWs (i.e., actual use plus missed opportunities for appropriate PPE use); and 5) stratification of HCW close contacts into medium- or low-risk groups for the purpose of recommending antiviral postexposure prophylaxis.

## Results

Nine tertiary-level university teaching hospitals in 3 states of eastern Australia participated in the study (Table 2). The simulations occurred in the winter season, from May through August 2006. All sites conducted targeted staff education sessions 1–2 weeks before their exercise. Seven of the 9 simulations proceeded for the planned 6 hours of observation, and 2 were curtailed because of a critical need for the emergency department bed. Had these latter 2 sites continued, the patient would almost certainly have spent the entire study period isolated in the emergency department, as suitable ward beds were not available. The time spent in each clinical area for each site is summarized in Table 2. All sites performed radiography within the emergency department.

**Table 2:** Participating institutions and time patient spent in each area*

Characteristic	Hospital								
	A	B	C	D	E	F	G	H	I
State	VIC	VIC	VIC	VIC	VIC	TAS	VIC	VIC	NSW
Urban/regional	Urban	Urban	Urban	Urban	Regional	Urban	Urban	Urban	Urban
Inpatient beds, no.	840	320	750	450	400	490	400	400	880
Annual admissions	67,700	40,000	79,500	47,200	61,200	52,300	45,300	93,100	71,600
Total simulation time, h	6	6	6	6	6	6	6	2.5	2.5
Triage time, h	0.3	0.3	0.1	0.1	0.1	0.1	0.1	0.2	0.1
ED time, h†	2	2.9	3	1.9	2.2	1.5	2.4	2.3‡	2.4‡
Ward time, h	3.7	2.8	3	3.9	3.7	4.4	3.5	–	–

*VIC, Victoria; TAS, Tasmania; NSW, New South Wales; ED, emergency department. †Includes time spent in ED radiology unit. ‡Simulation of avian or pandemic influenza ended prematurely because beds were needed.

The number of close contacts and total exposures to the potential API patient are summarized in Table 3. The highest number occurred in the first hour of hospital care (triage and emergency department), which correlated with the initial intensive clinical and radiologic assessment and specimen collection. Patient transfer between areas was another peak time for exposures. The average number of close contacts for each API patient during the study period was 12.3 (median 11, range 6–17), with 19.3 exposures (median 20, range 15–26). HCW close contacts constituted 85% of all close contacts; the remainder were patients or visitors who were generally exposed in the triage area.

**Table 3:** Number of close contacts (CCs) and exposures to API patient*

Characteristic	No. CCs (no. exposures) per hospital									
	A	B	C	D	E	F	G	H	I	Mean
Total	17 (26)	15 (20)	6 (15)	11 (20)	14 (17)	12 (20)	11 (17)	10 (11)†	6 (8)†	12.3 (19.3)‡
By clinical area										
Triage	8 (8)	4 (4)	1 (1)	2 (2)	5 (5)	1 (1)	3 (3)	7 (7)	3 (3)	3.8 (3.8)
ED	5 (11)	7 (11)	3 (9)	7 (10)	6 (9)	7 (10)	6 (9)	3 (4)†	3 (5)†	5.9 (9.9)‡
Ward	4 (7)	4 (5)	2 (5)	2 (8)	3 (3)	4 (9)	2 (5)	–	–	3.0 (6.0)
By study period, h										
0–1	10 (12)	8 (8)	3 (4)	6 (8)	7 (7)	8 (8)	5 (5)	9 (10)	5 (6)	6.8 (7.6)
1–2	2 (3)	0 (1)	1 (2)	2 (2)	3 (5)	2 (6)	3 (3)	0 (0)	1 (2)	1.6 (2.7)
2–3	2 (4)	0 (2)	0 (4)	3 (5)	2 (3)	1 (3)	0 (2)	1 (1)†	0 (0)†	1.1 (3.3)‡
3–4	3 (5)	5 (6)	2 (3)	0 (2)	0 (0)	1 (1)	2 (3)	–	–	1.9 (2.9)
4–5	0 (1)	2 (2)	0 (1)	0 (1)	1 (1)	0 (2)	1 (2)	–	–	0.6 (1.4)
5–6	0 (0)	0 (1)	0 (1)	0 (2)	1 (1)	0 (0)	0 (2)	–	–	0.1 (1.0)
By HCW status										
Non-HCW	3 (3)	5 (5)	0 (0)	0 (0)	3 (3)	0 (0)	2 (2)	4 (4)†	0 (0)†	1.9 (1.9)‡
HCW	14 (23)	10 (15)	6 (15)	11 (20)	11 (14)	12 (20)	9 (15)	6 (7)†	6 (8)†	10.4 (17.4)‡
No. HCW CCs (%) who wore complete PPE set during each exposure§	2	3	5	9	8	8	8	2†	3†	6.1 (59)‡
No. HCW CCs (%) who wore N95 masks during each exposure§	12	7	6	10	11	12	9	5†	3†	9.6 (92)‡

*API, avian (H5N1) or pandemic influenza; ED, emergency department; HCW, healthcare worker; PPE, personal protective equipment. †Incomplete data as simulation terminated after 2.5 h. ‡Excludes data from sites H and I. §World Health Organization recommendations (Table 1).

All 9 sites processed the respiratory specimen, with an average of 2.9 HCWs (median 3, range 2–6) handling or transporting the specimen, predominantly in the pathology department. Two sites used a vacuum transport system to deliver specimens from the emergency department to the laboratory, contrary to WHO recommendations (*7*).

Environmental decontamination of clinical areas after departure of the suspected API patient was performed haphazardly at all sites. The triage area was appropriately cleaned in none of the 9 sites, whereas the emergency department and ward areas at sites that completed the full simulation were cleaned appropriately in 6 of 7, and 4 of 7 instances, respectively; 1–2 cleaners were required per clinical area to appropriately perform this task.

Large quantities of N95 masks, disposable gowns, gloves, and eye protection were used and indicated during the study period (Table 4). Adherence to appropriate use by HCWs (HCW close contacts and ancillary HCWs) was variable and depended on the particular PPE item, clinical area, and participating institution. Appropriate use of N95 masks by HCWs occurred in 93% of exposures (actual use/total opportunities for PPE use, 18/19.4), although the corresponding figures for disposable gowns, gloves, and eye protection were lower (77%, 83%, and 73%, respectively).

**Table 4:** Actual and total opportunities for PPE item use by HCWs during the study period*

PPE item type	Actual PPE use (total opportunities for PPE item use) by hospital†										Compliance,¶ %
	A	B	C	D	E	F	G	H‡	I‡	Mean§	
N95 masks	20 (22)	11 (16)	18 (18)	20 (22)	16 (17)	23 (23)	18 (18)	6 (8)	8 (11)	18 (19.4)	93
Gowns	18 (29)	11 (18)	17 (21)	19 (24)	15 (17)	20 (25)	20 (21)	6 (11)	9 (12)	17.1 (22.1)	77
Gloves	27 (35)	12 (20)	18 (21)	21 (27)	19 (21)	23 (25)	26 (27)	8 (11)	10 (13)	20.9 (25.1)	83
Eye protection	4 (20)	4 (13)	14 (16)	18 (21)	14 (16)	21 (22)	17 (17)	3 (7)	4 (7)	13.1 (17.9)	73
Shoe protection#	–	4	2	–	9	–	–	–	1	2.1	
Hats#	–	1	13	–	14	–	–	–	5	4	

*PPE, personal protective equipment; HCWs, healthcare workers †See Table 1 for definitions. ‡Incomplete data as hospitals H and I terminated simulation after 2.5 h. §Excludes data from hospitals H and I. ¶Actual/total opportunities for PPE item use. #Use of shoe protection and hats not proscribed by the World Health Organization for routine use; data recorded only if these items were used.

HCW close contacts were stratified into either moderate- or low-risk groups, depending on whether an appropriate PPE set was worn during every exposure. The proportions of HCW close contacts who appropriately wore a PPE set, rather than an N95 mask alone, for every exposure were 59% and 92%, respectively. Thus, depending on how rigorously WHO antiviral medication guidelines (*9*) were followed, from 8% to 41% of all HCW close contacts would be classified as having experienced a medium-risk exposure and therefore would potentially require postexposure antiviral prophylaxis. This amounts to an average of 0.8 to 4.3 courses of antiviral medication per suspected API patient during the initial 6 hours of management.

## Discussion

To our knowledge, this is the first multicenter study to estimate the quantity of PPE and antiviral therapy that may be required to manage patients with suspected API admitted to hospitals. During the initial 6 hours of hospital assessment, the number of close contacts of a single suspected API patient was high (mean 12.3), with a mean number of exposures of 19.3. Not surprisingly, most (85%) close contacts were HCWs, and PPE use was at its most intense in the first hour of emergency department assessment. Our data suggest that in the initial 6 hours alone, HCWs managing suspected API case-patients would require ≈20–25 PPE sets (mean quantities: 19.4 N95 masks, 22.1 gowns, and 25.1 pairs of gloves). Although a high proportion of HCW close contacts (mean 92%) wore an N95 mask appropriately for all exposures, appropriate concomitant use of other PPE items was less (mean 59% of exposures). Even with the widespread availability of PPE, this observed inadequate utilization rate meant that from 8% to 41% of HCW close contacts were likely to require postexposure antiviral prophylaxis if current WHO recommendations were followed (*9*). If appropriate PPE, especially N95 masks, were not available, the number of HCWs who would experience moderate-risk API exposure requiring postexposure antiviral prophylaxis would increase substantially.

Notably, a substantial minority of close contacts (15%; ≈2 per API patient) were non-HCWs (e.g., hospital patients or visitors), generated primarily in the triage area. Although the duration of unprotected exposure was often short (<5 minutes) for these persons, they represent a potential risk for subsequent community and hospital spread of API. This highlights the importance, in triage and reception areas particularly, of using appropriate infection control measures and signage to assist in cohorting of potential API patients and minimizing exposure of unprotected bystanders.

The critical importance of effective PPE in hospital infection control was demonstrated during the outbreak of SARS in 2003 (*10*–*14*). Nosocomial transmission of SARS was a prominent feature of the epidemic (*15*) and played a large role in the initiation and maintenance of outbreaks. As reported in a case-control study by Seto et al. (*13*), staff who used masks (in particular), gowns, and performed hand hygiene were less likely to become SARS infected than those who did not. Similarly, Lau et al. (*14*) noted that inconsistent use of PPE by HCWs working on wards with SARS patients in Hong Kong was associated with a significantly higher risk for nosocomial disease transmission. Provision of adequate PPE stock is therefore likely to be important in controlling the spread of API.

Many countries are compiling extensive stockpiles of PPE and antiviral medications for use if a new pandemic occurs. Planning for sufficient numbers of resource items is complex and dependent on estimations of pandemic-related additional emergency presentations, hospitalizations, general practice, and outpatient visits. In Australia, official estimates of additional hospitalizations range from 57,900 to 148,000 (*4*). Our data suggest that management of this number of hospitalizations without regard for suspected influenza patients who are assessed but who are not sufficiently ill to require admission, would require from 1,123,260 to 3,714,800 PPE sets (depending on whether they were N95 masks, gowns, or gloves, or all 3 items). Although ascertaining (from these data) the number of courses of postexposure antiviral prophylaxis required is difficult, if stocks of readily available PPE were inadequate, the number of courses of antiviral medication required would likely increase dramatically, up to 12–13 courses per suspected API case during the initial 6-hour assessment. Thus, adequate stocks of PPE provide a means of protecting valuable antiviral drug stockpiles for use in ill or heavily exposed persons.

An important consideration when extrapolating our data to other healthcare systems is that recommendations regarding the optimal form of respiratory protection vary between countries. The WHO interim guidelines for management of human cases of avian influenza (AI) state, “HCWs working with AI-infected patients should select the highest level of respiratory protection available, preferably a particulate respirator… designed to protect the wearer from respiratory aerosols expelled by others” (*7*). This recommendation is reflected in the Australian pandemic influenza guidelines (*3*) and explains the high use of N95 masks in our study. However, pandemic influenza plans in the United Kingdom (*5*), United States (*6*), and Canada (*16*) currently recommend the use of surgical masks for close patient care, unless the HCW is engaged in procedures in which aerosolization occurs. Thus the proportion of N95 masks to surgical masks required will vary between countries with different guidelines, which affects assessment of stockpile adequacy. Our study did not assess the relative efficacy of N95 masks compared with surgical masks for protection against API transmission.

This study has several limitations. First, the duration of the study was short (6 hours), much shorter than the likely in-hospital stay of days for a patient with severe influenza. Thus, total PPE and antiviral agent usage per admission is likely to be substantially higher. Second, the study was conducted at a less busy time of day for emergency departments and therefore may not reflect the greater number of persons who would likely be exposed in the triage and emergency department areas during busier periods. Third, the patient was not clinically unwell or hypoxic; thus, relatively few HCWs were required to assess, manage, or review the API patient’s condition. Fourth, we observed the management of the index API case-patient alone, although we acknowledge that actual patients are likely to come to the hospital with other household members (high-risk close contacts). However, extending observation to include management of asymptomatic but potentially infectious accompanying persons in a standardized manner would have substantially increased the complexity of the exercise. Our findings, therefore, likely underestimate the true resources required and contacts exposed for the management of a genuine API patient. Finally, the presence of observers and the preceding education sessions may have artificially increased compliance with PPE use, although in the event of a true pandemic one might assume that HCW compliance rates would be high as they aim to minimize their personal risk. Also, this study was designed to quantify the use of PPE in an environment with raised awareness of infection control practice, mimicking that which might occur during a pandemic, and thus provide relevant data for health resource planners.

This study suggests that managing a single API patient is resource intensive and exposes a high number of persons to a potentially severe infection. These data represent the likely minimum clinical resources required during an API patient’s initial hospital assessment using current WHO-derived infection control guidelines. Given our findings, if a global influenza pandemic occurs with attack rates even on the lower end of projected estimates, demand for PPE and antiviral medication in healthcare facilities will likely outstrip current supply in industrialized countries, let alone the supply in resource-poor settings. Further studies are needed to assess resource usage in other healthcare settings such as intensive care units, fever clinics, general practice, and the community.

## References

[1] Meltzer MI, Cox NJ, Fukuda K. The economic impact of pandemic influenza in the United States: priorities for intervention. Emerg Infect Dis. 1999;5:659–71. 10.3201/eid0505.990507 10511522 PMC2627723

[2] World Health Organization Writing Group. Nonpharmaceutical interventions for pandemic influenza, national and community measures. Emerg Infect Dis. 2006;12:81–7. 16494723 10.3201/eid1201.051371 PMC3291415

[3] National Influenza Pandemic Action Committee. Interim infection control guidelines for pandemic influenza in healthcare and community settings. Annex to Australian health management plan for pandemic influenza, June 2006. [cited 2006 Jul 2]. Available from http://www.health.gov.au/internet/wcms/publishing.nsf/content/ohp-pandemic-infect-control-gl-toc.htm

[4] Department of Health and Ageing. Australian health management plan for pandemic influenza, May 2006. [cited 2006 May 15]. Available from http://www.health.gov.au/internet/wcms/publishing.nsf/content/ohp-pandemic-ahmppi.htm/$file/ahmppi-print.pdf

[5] Health Protection Agency. Guidance for pandemic influenza: infection control in hospitals and primary care settings, October 2005. [cited 2006 Oct 1]. Available from http://www.dh.gov.uk/assetroot/04/12/17/54/04121754.pdf

[6] United States Department of Health and Human Services. HHS pandemic influenza plan supplement 4, infection control, November 2006. [cited 2006 November 20]. Available from http://www.hhs.gov/pandemicflu/plan/sup4.html

[7] World Health Organization. Avian influenza, including influenza A (H5N1) in humans: WHO interim infection control guidelines for health care facilities, 2006. [cited 2006 May 15]. Available from www.who.int/csr/disease/avian_influenza/guidelines/infectioncontrol1/en

[8] Brown TL, Burrell LJ, Edmonds D, Martin R, O’Keeffe J, Johnson P, Hand hygiene: a standardized tool for assessing compliance. Australian Infection Control. 2005;10:1–6.

[9] World Health Organization. WHO rapid advice guidelines on pharmacological management of humans infected with avian influenza A (H5N1) virus, 2006. [cited 2006 Aug 20]. Available from http://www.who.int/medicines/publications/who_psm_par_2006.6.pdf

[10] Peiris JS, Yuen KY, Osterhaus AD, Stöhr K. The severe acute respiratory syndrome. N Engl J Med. 2003;349:2431–41. 10.1056/NEJMra032498 14681510

[11] Chan-Yeung M. Severe acute respiratory syndrome (SARS) and healthcare workers. Int J Occup Environ Health. 2004;10:421–7. 15702757 10.1179/oeh.2004.10.4.421

[12] Murphy C. The 2003 SARS outbreak: global challenges and innovative infection control measures. Online J Issues Nurs [serial on the internet]. 2006 Jan 31. [cited 2006 Sep 1]. Available from http://www.nursingworld.org/ojin/topic29/tpc29_5.htm 16629506

[13] Seto WH, Tsang D, Yung RWH, Ching TY, Ng TK, Ho M, Effectiveness of precautions against droplets and contact in prevention of nosocomial transmission of severe acute respiratory syndrome (SARS). Lancet. 2003;361:1519–20. 10.1016/S0140-6736(03)13168-6 12737864 PMC7112437

[14] Lau JT, Fung KS, Wong TW, Kim JH, Wong E, Chung S, SARS transmission among hospital workers in Hong Kong. Emerg Infect Dis. 2004;10:280–6. 15030698 10.3201/eid1002.030534 PMC3322933

[15] World Health Organization. Summary of probable SARS cases with onset of illness from 1 November 2002 to 31 July 2003 (based on data as of 31 December 2003). [cited 2006 Sep 1]. Available from http://www.who.int/csr/sars/country/table2004_04_21/en/index.html

[16] Public Health Agency of Canada. Infection control and occupational health guidelines during pandemic influenza in traditional and non-traditional health care settings (annex F), June 2006. [cited 2006 Oct 1]. Available from http://www.phac-aspc.gc.ca/cpip-pclcpi/pdf-e/15-cpip-appendix-f-infection-control_e.pdf

